# Tetra­kis(μ-5-bromo­nicotinato)-κ^3^ *O*,*O*′:*O*′;κ^3^ *O*:*O*,*O*′;κ^4^ *O*:*O*′-bis­[diaqua­(5-bromo­nicotinato-κ^2^ *O*,*O*′)neodymium(III)] dihydrate

**DOI:** 10.1107/S1600536811023798

**Published:** 2011-06-25

**Authors:** Jing Huang, Jin Zhang, Hong-Ji Chen

**Affiliations:** a Department of Chemistry, Jinan University, Guangzhou 510632, People’s Republic of China; b Department of Materials Science and Engineering, Jinan University, Guangzhou 510632, People’s Republic of China

## Abstract

In the title compound, [Nd_2_(C_6_H_3_BrNO_2_)_6_(H_2_O)_4_]·2H_2_O, the Nd^III^ ion is coordinated by nine O atoms from one chelating 5-bromo­nicotinate ligand, four bridging 5-bromo­nicotinate ligands and two water mol­ecules, exhibiting a distorted three-capped triangular-prismatic geometry. Two Nd^III^ ions are bridged by four carboxyl­ate groups in bi- and tridentate modes, forming a centrosymmetric dinuclear unit, with an Nd⋯Nd distance of 4.0021 (5) Å, and intra­molecular π–π inter­actions between the pyridine rings [centroid–centroid distance = 3.960 (2) Å]. Inter­molecular π–π inter­actions [centroid–centroid distances = 3.820 (2) and 3.804 (2) Å] and O—H⋯N and O—H⋯O hydrogen bonds connect the dinuclear mol­ecules into a three-dimensional supra­molecular network.

## Related literature

For general background to lanthanide complexes with carboxyl­ates, see: Ragunathan & Schneider (1996 ▶); Shibasaki & Yoshikawa (2002 ▶). For dimeric lanthanide carboxyl­ates, see: Rupam *et al.* (2010 ▶); Song *et al.* (2004 ▶); Yang & Chen (2009 ▶).
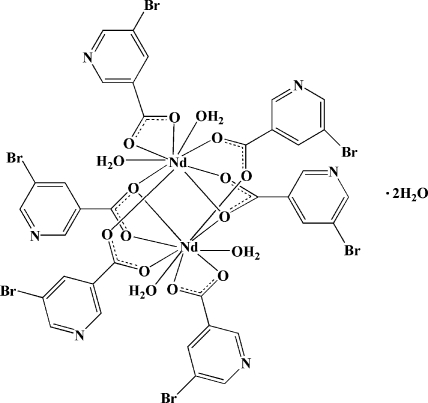


## Experimental

### 

#### Crystal data

[Nd_2_(C_6_H_3_BrNO_2_)_6_(H_2_O)_4_]·2H_2_O
*M* *_r_* = 1602.60
Monoclinic, 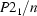

*a* = 11.5278 (13) Å
*b* = 16.6616 (18) Å
*c* = 12.2711 (13) Å
β = 102.478 (2)°
*V* = 2301.3 (4) Å^3^
*Z* = 2
Mo *K*α radiation
μ = 7.52 mm^−1^
*T* = 110 K
0.42 × 0.38 × 0.36 mm

#### Data collection

Bruker APEX CCD diffractometer
Absorption correction: multi-scan (*SADABS*; Sheldrick, 1996 ▶) *T*_min_ = 0.059, *T*_max_ = 0.067
11552 measured reflections
4999 independent reflections
4295 reflections with *I* > 2σ(*I*)
*R*_int_ = 0.023

#### Refinement

*R*[*F* ^2^ > 2σ(*F* ^2^)] = 0.022
*wR*(*F* ^2^) = 0.054
*S* = 1.07
4999 reflections
331 parameters
6 restraints
H atoms treated by a mixture of independent and constrained refinement
Δρ_max_ = 1.02 e Å^−3^
Δρ_min_ = −1.07 e Å^−3^

### 

Data collection: *SMART* (Bruker, 2007 ▶); cell refinement: *SAINT* (Bruker, 2007 ▶); data reduction: *SAINT*; program(s) used to solve structure: *SHELXTL* (Sheldrick, 2008 ▶); program(s) used to refine structure: *SHELXTL*; molecular graphics: *DIAMOND* (Brandenburg, 1999 ▶); software used to prepare material for publication: *SHELXTL*.

## Supplementary Material

Crystal structure: contains datablock(s) I, global. DOI: 10.1107/S1600536811023798/hy2440sup1.cif

Structure factors: contains datablock(s) I. DOI: 10.1107/S1600536811023798/hy2440Isup2.hkl

Additional supplementary materials:  crystallographic information; 3D view; checkCIF report

## Figures and Tables

**Table 1:** Hydrogen-bond geometry (Å, °)

*D*—H⋯*A*	*D*—H	H⋯*A*	*D*⋯*A*	*D*—H⋯*A*
O7—H71⋯N3^i^	0.84 (1)	1.94 (2)	2.769 (3)	165 (5)
O7—H72⋯O9^ii^	0.85 (3)	1.97 (2)	2.780 (3)	160 (4)
O8—H81⋯O9^ii^	0.85 (1)	1.87 (1)	2.699 (3)	164 (3)
O8—H82⋯N2^iii^	0.85 (1)	1.88 (1)	2.735 (3)	175 (5)
O9—H91⋯N1	0.85 (3)	1.96 (3)	2.801 (3)	172 (4)
O9—H92⋯O4^iii^	0.84 (1)	2.21 (2)	2.981 (3)	153 (4)
O9—H92⋯O8^iii^	0.84 (1)	2.37 (4)	2.989 (3)	131 (4)

Symmetry codes: (i) 
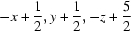
; (ii) 
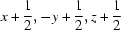
; (iii) 
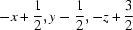
.

**Fig. 1.**
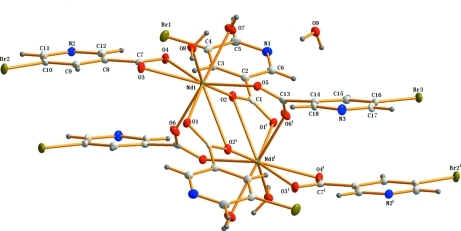
The molecular structure of the title compound, showing displacement ellipsoids at the 50% probability level. [Symmetry code: (i) -x, 1-y, 2-z.]

**Fig. 2.**
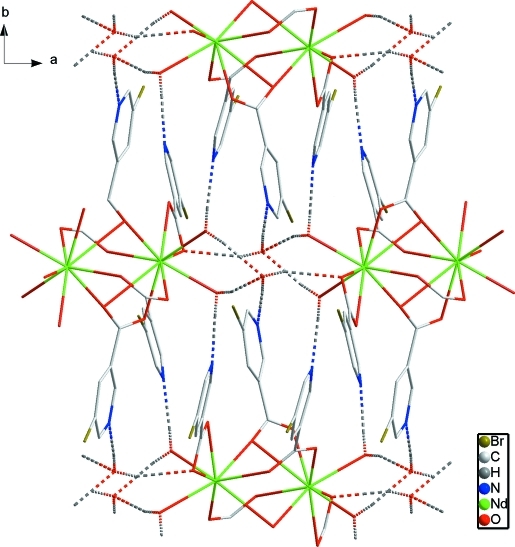
Hydrogen-bonded network in the title compound. Dashed lines denote hydrogen bonds.

## supplementary crystallographic information

### Comment

Part of attentions has been paid to rational design and synthesis of lanthanide carboxylates due to their structural diversity and potential applications as catalysts (Ragunathan & Schneider, 1996; Shibasaki & Yoshikawa, 2002). Recent research results showed that olefin epoxidation reaction can be catalyzed by dimeric lanthanide carboxylates (Rupam *et al.*, 2010). A few crystal structures of dimeric lanthanide carboxylates from 5-bromonicotinic acid (5-BrnicH) ligand, such as [La(5-Brnic)_3_(H_2_O)_2_]_2_.H_2_O, [Gd(5-Brnic)_3_(H_2_O)_2_]_2_ (Rupam *et al.*, 2010) and [Sm(5-Brnic)_3_(H_2_O)_2_]_2_.H_2_O (Song *et al.*, 2004), have been reported. As the research interest in the catalytic behavior of lanthanide compounds and the easy formation of dinuclear unit by using 5-BrnicH ligand (Yang & Chen, 2009), we synthesized a lanthanide carboxylate, the title compound. We report here its crystal structure.

The title compound is a dimeric lanthanide carboxylate that contains two Nd^III^ ions, six 5-Brnic ligands and four coordinated and two uncoordinated water molecules (Fig. 1). In the dimer, each Nd^III^ ion is nine-coordinated by nine O atoms, two of them from one (κ^2^O,O')-carboxylate group, two from two (κ^2^O:O')-carboxylate groups, three from two (κ^3^O,O':O')-carboxylate groups, and two from the coordinated water molecules. The coordination environment of the metal atom can be described as distorted three-capped triangular-prismatic. The two Nd^III^ ions are bridged by four carboxylate groups in bi- and tridentate modes, forming a centrosymmetric dinuclear unit, with a Nd···Nd distance of 4.0021 (5) Å and intramolecular π–π interactions between the pyridine rings [centroid–centroid distance = 3.960 (2) Å]. Intermolecular π–π interactions [centroid–centroid distances = 3.820 (2) and 3.804 (2) Å] and O—H···N and O—H···O hydrogen bonds (Table 1) connect the dinuclear molecules into a three-dimensional supramolecular network (Fig. 2).

In addition, the nine-coordinated Nd^III^ ion in the title compound exhibits higher coordination number than that in the dimers [Gd(5-Brnic)_3_(H_2_O)_2_]_2_ (Rupam *et al.*, 2010) and [Sm(5-Brnic)_3_(H_2_O)_2_]_2_.H_2_O (Song *et al.*, 2004). As Nd^III^ ion has bigger ionic radius than that of Gd^III^ and Sm^III^ ions, we owe the increasing in coordination number to the principle of lanthanide contraction.

### Experimental

A mixture of neodymium oxide (0.2 mmol, 0.067 g), 5-bromonicotinic acid (0.5 mmol, 0.101 g) and 10 ml water was sealed in a 15 ml Teflon-line autoclave and heated to 363 K for 72 h. The reaction solution was then cooled down to room temperature at a rate of 5 K per hour. Brown single crystals suitable for X-ray crystallography analysis were obtained (yield: 42%). Analysis, calculated for C_18_H_15_Br_3_N_3_NdO_9_: C 26.38, H 1.89, N 5.24%; found: C 26.17, H 1.98, N 5.41%. IR (cm^-1^, KBr): 3403 bm, 3051 m, 1618 vs, 1550 s, 1541 s, 1442 vs, 1400 vs, 1292 m, 1186 vw, 1130 w, 1026 w, 953 vw, 891 vw, 881 w, 789 m, 741 m, 687 m, 442 m.

### Refinement

H atoms bonded to C atoms were positioned geometrically and refined as riding atoms, with C—H = 0.95 Å and with *U*_iso_(H) = 1.2*U*_eq_(C). H atoms of water molecules were located from a difference Fourier map and refined isotropically, with a distance restraint of O—H = 0.85 (1) Å. The highest residual electron density was found at 0.86 Å from Nd1 atom and the deepest hole at 0.79 Å from Nd1 atom.

### Crystal data

**Table:**

[Nd_2_(C_6_H_3_BrNO_2_)_6_(H_2_O)_4_]·2H_2_O	*F*(000) = 1524
*M**_r_* = 1602.60	*D*_x_ = 2.313 Mg m^−^^3^
Monoclinic, *P*2_1_/*n*	Mo *K*α radiation, λ = 0.71073 Å
Hall symbol: -P 2yn	Cell parameters from 4999 reflections
*a* = 11.5278 (13) Å	θ = 2.5–27.1°
*b* = 16.6616 (18) Å	µ = 7.52 mm^−^^1^
*c* = 12.2711 (13) Å	*T* = 110 K
β = 102.478 (2)°	Block, brown
*V* = 2301.3 (4) Å^3^	0.42 × 0.38 × 0.36 mm
*Z* = 2	

### Data collection

**Table:**

Bruker APEX CCD diffractometer	4999 independent reflections
Radiation source: fine-focus sealed tube	4295 reflections with *I* > 2σ(*I*)
graphite	*R*_int_ = 0.023
φ and ω scans	θ_max_ = 27.1°, θ_min_ = 2.1°
Absorption correction: multi-scan (*SADABS*; Sheldrick, 1996)	*h* = −11→14
*T*_min_ = 0.059, *T*_max_ = 0.067	*k* = −21→19
11552 measured reflections	*l* = −15→15

### Refinement

**Table:**

Refinement on *F*^2^	Primary atom site location: structure-invariant direct methods
Least-squares matrix: full	Secondary atom site location: difference Fourier map
*R*[*F*^2^ > 2σ(*F*^2^)] = 0.022	Hydrogen site location: inferred from neighbouring sites
*wR*(*F*^2^) = 0.054	H atoms treated by a mixture of independent and constrained refinement
*S* = 1.07	*w* = 1/[σ^2^(*F*_o_^2^) + (0.0266*P*)^2^] where *P* = (*F*_o_^2^ + 2*F*_c_^2^)/3
4999 reflections	(Δ/σ)_max_ = 0.066
331 parameters	Δρ_max_ = 1.02 e Å^−^^3^
6 restraints	Δρ_min_ = −1.07 e Å^−^^3^

### Fractional atomic coordinates and isotropic or equivalent isotropic displacement parameters (Å^2^)

**Table:**

	*x*	*y*	*z*	*U*_iso_*/*U*_eq_	
Br1	−0.09938 (3)	0.096820 (19)	1.12865 (3)	0.01690 (8)	
Br2	0.24543 (3)	0.605255 (19)	0.35014 (3)	0.01538 (8)	
Br3	0.08252 (3)	0.38137 (2)	1.62068 (3)	0.01714 (8)	
C1	0.0190 (2)	0.35415 (17)	0.9159 (2)	0.0080 (6)	
C2	−0.0119 (2)	0.26663 (17)	0.9148 (2)	0.0091 (6)	
C3	−0.0484 (3)	0.23220 (18)	1.0047 (2)	0.0100 (6)	
H3	−0.0621	0.2641	1.0648	0.012*	
C4	−0.0640 (3)	0.15010 (18)	1.0039 (3)	0.0120 (6)	
C5	−0.0505 (3)	0.10527 (18)	0.9120 (3)	0.0143 (7)	
H5	−0.0654	0.0492	0.9114	0.017*	
C6	0.0039 (3)	0.21785 (18)	0.8270 (3)	0.0123 (6)	
H6	0.0306	0.2417	0.7665	0.015*	
C7	0.2081 (3)	0.60360 (18)	0.7791 (2)	0.0108 (6)	
C8	0.2037 (3)	0.64877 (18)	0.6724 (2)	0.0102 (6)	
C9	0.2253 (3)	0.60917 (18)	0.5792 (2)	0.0105 (6)	
H9	0.2431	0.5535	0.5814	0.013*	
C10	0.2200 (3)	0.65385 (18)	0.4825 (2)	0.0103 (6)	
C11	0.1923 (3)	0.73465 (18)	0.4816 (3)	0.0134 (6)	
H11	0.1892	0.7644	0.4150	0.016*	
C12	0.1762 (3)	0.72998 (18)	0.6646 (3)	0.0119 (6)	
H12	0.1611	0.7566	0.7286	0.014*	
C13	0.1084 (3)	0.42457 (17)	1.1932 (2)	0.0107 (6)	
C14	0.1252 (3)	0.37044 (18)	1.2944 (2)	0.0100 (6)	
C15	0.0977 (3)	0.39880 (18)	1.3926 (2)	0.0107 (6)	
H15	0.0718	0.4525	1.3980	0.013*	
C16	0.1090 (3)	0.34655 (19)	1.4820 (2)	0.0110 (6)	
C17	0.1423 (3)	0.26774 (18)	1.4697 (2)	0.0130 (6)	
H17	0.1485	0.2323	1.5313	0.016*	
C18	0.1593 (3)	0.29081 (18)	1.2895 (2)	0.0106 (6)	
H18	0.1789	0.2718	1.2228	0.013*	
H71	0.313 (4)	0.6236 (10)	1.119 (4)	0.063 (16)*	
H72	0.368 (2)	0.548 (2)	1.133 (3)	0.033 (12)*	
H81	0.395 (2)	0.4366 (19)	1.0285 (15)	0.010 (9)*	
H82	0.340 (4)	0.3837 (7)	0.958 (4)	0.066 (17)*	
H91	−0.001 (3)	0.0648 (16)	0.704 (2)	0.026 (11)*	
H92	0.0725 (15)	0.017 (3)	0.656 (4)	0.052 (15)*	
N1	−0.0172 (2)	0.13855 (15)	0.8243 (2)	0.0146 (6)	
N2	0.1699 (2)	0.77251 (15)	0.5710 (2)	0.0126 (5)	
N3	0.1659 (2)	0.23932 (15)	1.3751 (2)	0.0142 (6)	
Nd1	0.157605 (13)	0.506682 (9)	0.957077 (13)	0.00682 (5)	
O1	0.10185 (18)	0.37520 (12)	0.87133 (17)	0.0113 (4)	
O2	0.03578 (18)	0.59795 (12)	1.03191 (17)	0.0118 (4)	
O3	0.18480 (19)	0.64042 (12)	0.86189 (17)	0.0138 (5)	
O4	0.23363 (18)	0.53005 (12)	0.78226 (17)	0.0126 (4)	
O5	0.17948 (19)	0.41883 (13)	1.12916 (18)	0.0150 (5)	
O6	−0.02224 (19)	0.52807 (13)	0.81744 (17)	0.0144 (5)	
O7	0.3080 (2)	0.57471 (14)	1.1001 (2)	0.0164 (5)	
O8	0.34606 (19)	0.43444 (13)	0.96599 (18)	0.0116 (4)	
O9	0.0016 (2)	0.02805 (14)	0.65696 (19)	0.0140 (5)	

### Atomic displacement parameters (Å^2^)

**Table:**

	*U*^11^	*U*^22^	*U*^33^	*U*^12^	*U*^13^	*U*^23^
Br1	0.02332 (18)	0.01014 (16)	0.01978 (17)	0.00051 (12)	0.01028 (14)	0.00525 (13)
Br2	0.02280 (17)	0.01451 (17)	0.01077 (15)	−0.00214 (12)	0.00791 (12)	−0.00125 (12)
Br3	0.01782 (16)	0.02518 (19)	0.00977 (15)	−0.00330 (13)	0.00597 (12)	−0.00356 (13)
C1	0.0111 (14)	0.0073 (14)	0.0042 (13)	0.0020 (11)	−0.0014 (11)	0.0000 (11)
C2	0.0093 (14)	0.0054 (14)	0.0124 (15)	0.0008 (11)	0.0015 (12)	0.0000 (12)
C3	0.0099 (14)	0.0086 (15)	0.0124 (15)	0.0018 (11)	0.0047 (12)	−0.0013 (12)
C4	0.0102 (15)	0.0105 (15)	0.0159 (16)	0.0010 (11)	0.0043 (12)	0.0040 (13)
C5	0.0166 (16)	0.0051 (15)	0.0207 (17)	−0.0013 (12)	0.0033 (13)	−0.0007 (13)
C6	0.0120 (15)	0.0119 (16)	0.0129 (15)	−0.0012 (12)	0.0023 (12)	−0.0013 (12)
C7	0.0089 (14)	0.0108 (15)	0.0129 (15)	−0.0020 (11)	0.0029 (12)	0.0026 (12)
C8	0.0113 (15)	0.0081 (15)	0.0109 (15)	−0.0023 (11)	0.0015 (12)	0.0016 (12)
C9	0.0102 (14)	0.0101 (15)	0.0116 (15)	−0.0011 (11)	0.0029 (12)	−0.0011 (12)
C10	0.0117 (15)	0.0107 (15)	0.0080 (14)	−0.0032 (11)	0.0011 (12)	−0.0016 (12)
C11	0.0171 (16)	0.0118 (16)	0.0115 (15)	−0.0028 (12)	0.0038 (12)	0.0029 (12)
C12	0.0116 (15)	0.0119 (16)	0.0123 (15)	−0.0017 (12)	0.0031 (12)	−0.0011 (12)
C13	0.0150 (15)	0.0062 (15)	0.0092 (15)	−0.0059 (12)	−0.0012 (12)	0.0008 (12)
C14	0.0089 (14)	0.0089 (15)	0.0116 (15)	−0.0013 (11)	0.0011 (12)	0.0017 (12)
C15	0.0122 (15)	0.0073 (15)	0.0125 (15)	0.0002 (11)	0.0027 (12)	−0.0008 (12)
C16	0.0109 (15)	0.0143 (16)	0.0085 (14)	−0.0028 (12)	0.0035 (12)	−0.0013 (12)
C17	0.0169 (16)	0.0126 (16)	0.0091 (15)	0.0001 (12)	0.0018 (12)	0.0060 (12)
C18	0.0137 (15)	0.0111 (16)	0.0069 (14)	−0.0003 (11)	0.0019 (12)	−0.0006 (12)
N1	0.0177 (14)	0.0087 (13)	0.0179 (14)	−0.0015 (10)	0.0051 (11)	−0.0043 (11)
N2	0.0169 (13)	0.0088 (13)	0.0122 (13)	−0.0023 (10)	0.0030 (11)	0.0025 (10)
N3	0.0193 (14)	0.0095 (13)	0.0137 (14)	0.0029 (10)	0.0034 (11)	0.0044 (11)
Nd1	0.01072 (8)	0.00413 (8)	0.00615 (8)	0.00027 (6)	0.00303 (6)	0.00031 (6)
O1	0.0151 (11)	0.0077 (11)	0.0124 (11)	−0.0005 (8)	0.0059 (9)	0.0002 (8)
O2	0.0175 (11)	0.0081 (11)	0.0105 (10)	0.0025 (9)	0.0046 (9)	0.0008 (9)
O3	0.0235 (12)	0.0086 (11)	0.0110 (11)	−0.0032 (9)	0.0074 (9)	−0.0013 (9)
O4	0.0197 (11)	0.0083 (11)	0.0113 (11)	0.0020 (9)	0.0064 (9)	0.0023 (9)
O5	0.0155 (11)	0.0180 (12)	0.0112 (11)	−0.0039 (9)	0.0020 (9)	0.0056 (9)
O6	0.0198 (12)	0.0088 (11)	0.0120 (11)	0.0027 (9)	−0.0026 (9)	−0.0002 (9)
O7	0.0201 (13)	0.0080 (12)	0.0187 (12)	0.0007 (9)	−0.0009 (10)	−0.0038 (10)
O8	0.0137 (11)	0.0078 (12)	0.0124 (11)	0.0004 (9)	0.0009 (9)	−0.0017 (9)
O9	0.0157 (12)	0.0138 (12)	0.0128 (12)	0.0015 (9)	0.0039 (10)	−0.0042 (9)

### Geometric parameters (Å, °)

**Table:**

Br1—C4	1.888 (3)	C13—O5	1.255 (4)
Br2—C10	1.894 (3)	C13—C14	1.513 (4)
Br3—C16	1.884 (3)	C14—C18	1.389 (4)
C1—O1	1.249 (3)	C14—C15	1.393 (4)
C1—O2^i^	1.273 (3)	C15—C16	1.385 (4)
C1—C2	1.500 (4)	C15—H15	0.9500
C2—C3	1.386 (4)	C16—C17	1.385 (4)
C2—C6	1.393 (4)	C17—N3	1.335 (4)
C3—C4	1.380 (4)	C17—H17	0.9500
C3—H3	0.9500	C18—N3	1.345 (4)
C4—C5	1.389 (4)	C18—H18	0.9500
C5—N1	1.340 (4)	Nd1—O2	2.384 (2)
C5—H5	0.9500	Nd1—O6	2.414 (2)
C6—N1	1.342 (4)	Nd1—O1	2.455 (2)
C6—H6	0.9500	Nd1—O7	2.461 (2)
C7—O4	1.259 (3)	Nd1—O8	2.465 (2)
C7—O3	1.264 (4)	Nd1—O4	2.517 (2)
C7—C8	1.501 (4)	Nd1—O5	2.537 (2)
C8—C9	1.389 (4)	Nd1—O3	2.566 (2)
C8—C12	1.388 (4)	Nd1—O2^i^	2.856 (2)
C9—C10	1.390 (4)	Nd1—Nd1^i^	4.0022 (5)
C9—H9	0.9500	O7—H71	0.84 (1)
C10—C11	1.383 (4)	O7—H72	0.85 (3)
C11—N2	1.338 (4)	O8—H81	0.85 (1)
C11—H11	0.9500	O8—H82	0.85 (1)
C12—N2	1.338 (4)	O9—H91	0.85 (3)
C12—H12	0.9500	O9—H92	0.84 (1)
C13—O6^i^	1.253 (4)		
			
O1—C1—O2^i^	123.6 (3)	O7—Nd1—O8	73.44 (7)
O1—C1—C2	118.1 (3)	O2—Nd1—O4	124.90 (7)
O2^i^—C1—C2	118.1 (3)	O6—Nd1—O4	77.05 (7)
O1—C1—Nd1	53.38 (14)	O1—Nd1—O4	83.10 (7)
O2^i^—C1—Nd1	71.76 (16)	O7—Nd1—O4	102.44 (8)
C2—C1—Nd1	161.08 (19)	O8—Nd1—O4	69.36 (7)
C3—C2—C6	119.1 (3)	O2—Nd1—O5	90.48 (7)
C3—C2—C1	120.5 (3)	O6—Nd1—O5	126.28 (7)
C6—C2—C1	120.2 (3)	O1—Nd1—O5	79.14 (7)
C4—C3—C2	117.8 (3)	O7—Nd1—O5	75.17 (7)
C4—C3—H3	121.1	O8—Nd1—O5	75.71 (7)
C2—C3—H3	121.1	O4—Nd1—O5	143.95 (7)
C3—C4—C5	120.1 (3)	O2—Nd1—O3	76.21 (7)
C3—C4—Br1	120.8 (2)	O6—Nd1—O3	73.70 (7)
C5—C4—Br1	119.1 (2)	O1—Nd1—O3	128.77 (7)
N1—C5—C4	122.2 (3)	O7—Nd1—O3	77.78 (7)
N1—C5—H5	118.9	O8—Nd1—O3	104.54 (7)
C4—C5—H5	118.9	O4—Nd1—O3	51.57 (7)
N1—C6—C2	122.7 (3)	O5—Nd1—O3	151.64 (7)
N1—C6—H6	118.7	O2—Nd1—O2^i^	80.82 (7)
C2—C6—H6	118.7	O6—Nd1—O2^i^	64.17 (7)
O4—C7—O3	122.4 (3)	O1—Nd1—O2^i^	48.76 (6)
O4—C7—C8	118.6 (3)	O7—Nd1—O2^i^	133.17 (7)
O3—C7—C8	119.0 (3)	O8—Nd1—O2^i^	112.90 (7)
O4—C7—Nd1	60.93 (15)	O4—Nd1—O2^i^	123.63 (6)
O3—C7—Nd1	63.19 (15)	O5—Nd1—O2^i^	63.04 (6)
C8—C7—Nd1	165.9 (2)	O3—Nd1—O2^i^	136.46 (6)
C9—C8—C12	119.0 (3)	O2—Nd1—C7	99.62 (8)
C9—C8—C7	120.1 (3)	O6—Nd1—C7	70.22 (8)
C12—C8—C7	120.9 (3)	O1—Nd1—C7	104.64 (8)
C8—C9—C10	117.7 (3)	O7—Nd1—C7	93.22 (8)
C8—C9—H9	121.1	O8—Nd1—C7	89.25 (8)
C10—C9—H9	121.1	O4—Nd1—C7	25.93 (7)
C11—C10—C9	119.7 (3)	O5—Nd1—C7	163.03 (8)
C11—C10—Br2	119.1 (2)	O3—Nd1—C7	26.09 (7)
C9—C10—Br2	121.1 (2)	O2^i^—Nd1—C7	131.83 (7)
N2—C11—C10	122.5 (3)	O2—Nd1—C1	105.66 (8)
N2—C11—H11	118.8	O6—Nd1—C1	70.10 (7)
C10—C11—H11	118.8	O1—Nd1—C1	24.09 (7)
N2—C12—C8	123.0 (3)	O7—Nd1—C1	141.37 (8)
N2—C12—H12	118.5	O8—Nd1—C1	91.49 (7)
C8—C12—H12	118.5	O4—Nd1—C1	105.05 (7)
O6^i^—C13—O5	126.3 (3)	O5—Nd1—C1	66.61 (7)
O6^i^—C13—C14	114.8 (3)	O3—Nd1—C1	140.85 (7)
O5—C13—C14	118.9 (3)	O2^i^—Nd1—C1	25.04 (7)
C18—C14—C15	118.7 (3)	C7—Nd1—C1	122.56 (8)
C18—C14—C13	121.4 (3)	O2—Nd1—Nd1^i^	44.79 (5)
C15—C14—C13	119.7 (3)	O6—Nd1—Nd1^i^	60.37 (5)
C16—C15—C14	118.2 (3)	O1—Nd1—Nd1^i^	83.95 (5)
C16—C15—H15	120.9	O7—Nd1—Nd1^i^	112.19 (6)
C14—C15—H15	120.9	O8—Nd1—Nd1^i^	143.38 (5)
C17—C16—C15	119.4 (3)	O4—Nd1—Nd1^i^	137.40 (5)
C17—C16—Br3	119.5 (2)	O5—Nd1—Nd1^i^	71.42 (5)
C15—C16—Br3	121.1 (2)	O3—Nd1—Nd1^i^	112.04 (5)
N3—C17—C16	123.0 (3)	O2^i^—Nd1—Nd1^i^	36.03 (4)
N3—C17—H17	118.5	C7—Nd1—Nd1^i^	125.13 (6)
C16—C17—H17	118.5	C1—Nd1—Nd1^i^	60.94 (6)
N3—C18—C14	123.0 (3)	C1—O1—Nd1	102.53 (17)
N3—C18—H18	118.5	C1^i^—O2—Nd1	172.35 (19)
C14—C18—H18	118.5	C1^i^—O2—Nd1^i^	83.20 (17)
C5—N1—C6	118.0 (3)	Nd1—O2—Nd1^i^	99.18 (7)
C12—N2—C11	118.0 (3)	C7—O3—Nd1	90.72 (17)
C17—N3—C18	117.7 (3)	C7—O4—Nd1	93.14 (17)
O2—Nd1—O6	72.07 (7)	C13—O5—Nd1	121.09 (19)
O2—Nd1—O1	127.79 (7)	C13^i^—O6—Nd1	135.0 (2)
O6—Nd1—O1	73.88 (7)	Nd1—O7—H71	129 (3)
O2—Nd1—O7	79.30 (8)	Nd1—O7—H72	118 (3)
O6—Nd1—O7	143.42 (7)	H71—O7—H72	113 (4)
O1—Nd1—O7	142.70 (7)	Nd1—O8—H81	115 (2)
O2—Nd1—O8	151.76 (7)	Nd1—O8—H82	115 (3)
O6—Nd1—O8	135.81 (7)	H81—O8—H82	100 (4)
O1—Nd1—O8	74.33 (7)	H91—O9—H92	110 (4)

Symmetry codes: (i) −*x*, −*y*+1, −*z*+2.

### Hydrogen-bond geometry (Å, °)

**Table:**

*D*—H···*A*	*D*—H	H···*A*	*D*···*A*	*D*—H···*A*
O7—H71···N3^ii^	0.84 (1)	1.94 (2)	2.769 (3)	165 (5)
O7—H72···O9^iii^	0.85 (3)	1.97 (2)	2.780 (3)	160 (4)
O8—H81···O9^iii^	0.85 (1)	1.87 (1)	2.699 (3)	164 (3)
O8—H82···N2^iv^	0.85 (1)	1.88 (1)	2.735 (3)	175 (5)
O9—H91···N1	0.85 (3)	1.96 (3)	2.801 (3)	172 (4)
O9—H92···O4^iv^	0.84 (1)	2.21 (2)	2.981 (3)	153 (4)
O9—H92···O8^iv^	0.84 (1)	2.37 (4)	2.989 (3)	131 (4)

Symmetry codes: (ii) −*x*+1/2, *y*+1/2, −*z*+5/2; (iii) *x*+1/2, −*y*+1/2, *z*+1/2; (iv) −*x*+1/2, *y*−1/2, −*z*+3/2.
